# Mutations Disrupting Histone Methylation Have Different Effects on Replication Timing in *S. pombe* Centromere

**DOI:** 10.1371/journal.pone.0061464

**Published:** 2013-05-01

**Authors:** Pao-Chen Li, Marc D. Green, Susan L. Forsburg

**Affiliations:** Molecular & Computational Biology Program, University of Southern California, Los Angeles, California, United States of America; St Jude Children's Research Hospital, United States of America

## Abstract

The fission yeast pericentromere comprises repetitive sequence elements packaged into heterchromatin marked by histone H3K9 methylation and Swi6 binding. Transient disruption of Swi6 during S phase allows a period of RNA synthesis which programs the RNAi machinery to maintain histone methylation. However, Swi6 is also required for early replication timing. We show that not only Swi6 but also the chromodomain protein Chp1 are delocalized during S phase. Different from loss of *swi6*, mutations that disrupt histone methylation in the centromere, *chp1*Δ and *clr4*Δ, undergo early DNA replication. However, timing is modestly delayed in RNAi mutants *dcr1*Δ or *rdp1*Δ, while *hrr1*Δ mutants resemble *swi6*Δ in their replication delay. Finally, we show that recruitment of RNA polymerase II in the centromere occurs independently of replication. These different effects indicate that replication timing is not simply linked to histone methylation.

## Introduction

In eukaryotic cells, replication origins fire at different times in S phase depending on their position and local chromatin structure, and availability of limiting replication factors (reviewed in [1]–[3]). In general, highly transcribed euchromatic regions replicate early, while transcriptionally repressed heterochromatin regions usually replicate late in S phase. However, in *S. pombe*, the repetitive pericentromeric heterochromatin replicates early, in contrast to telomeres [4]–[6]. All these domains are characterized by binding of the Heterochromatin Protein 1 (HP1) homologue Swi6 to methylated histone H3K9 [7].

The heterochromatin in the *S. pombe* pericentromere is transiently transcribed during G1 and S phase, after Swi6 is dislodged from the chromatin by phosphorylation of the H3S10 residue by the mitotic Aurora kinase [8], [9]. Loss of Swi6 provides an opportunity for RNA polymerase II to synthesize centromere transcripts bidirectionally in the pericentromeric region during S phase. The RNAs are amplified by Rdp1-dependent RNA Polymerase Complex (RDRC) to generate double stranded RNAs that are further processed to siRNAs by Dcr1. These RNAs together with Ago1, Chp1 and Tas3 form a RNA-induced-transcriptional-gene-silencing complex (RITS) that targets nascent repeat transcripts at the centromere. RITS recruits histone methyltranferase Clr4 to methylate H3K9, which re-establishes Swi6 binding [10], [11]. However, recent studies show that Swi6 also acts upstream of the RDRC, via interaction with the Ers1 [12]–[14].

Previous studies have shown that early replication timing of the *S. pombe* pericentromere depends on Swi6; in its absence, the pericentromere replicates very late [4], [15]. Swi6 associates with the replication initiating DDK complex, consisting of the Hsk1^Cdc7^ kinase and its regulatory subunit Dfp1^Dbf4^ [4], [16]. The pre-replication complex protein Cdc18^Cdc6^ also interacts with Swi6 to affect replication timing in this domain [15]. Deletion of the histone methyltransferase Clr4 suppresses the late replication caused by *swi6*Δ, as does artificial tethering of the DDK complex to the chromatin [4]. This is consistent with observations suggesting that DDK is a limiting factor for replication [17]. These results suggest that Swi6 counters the late replication of heterochromatin by recruiting or enriching limiting replication initiation proteins in this region.

Interestingly, histone methylation in the pericentromeric heterochromatin is also linked to replication fork proteins including DNA polymerase alpha and epsilon, possibly providing a means of maintenance during replication [18], [19]. Recent studies suggest that replication and transcription in this domain must be coordinated to prevent polymerase collisions and replication fork breakdown [20]. Loss of heterochromatin makes the cells particularly vulnerable to disruptions in replication fork stability [20], [21].

Together, these data suggest a paradox in the effect of Swi6 on replication timing in the centromere heterochromatin. On the one hand, studies suggest that Swi6 is largely dislodged during mitosis and only recruited back later in S phase, allowing a window of transcription [8], [22] while on the other, Swi6 is clearly required for early replication initiation in this domain [4], [15] and acts upstream of RDRC [12]–[14]. This suggests that there are distinct roles for Swi6: one important for replication, possibly via interactions with the pre-replication complex (e.g., Cdc18) and the DDK kinase, and a second which is necessary for re-establishment of the silent heterochromatin, possibly via Swi6 interactions with CAF1 and PCNA (e.g., [23]).

Although there are numerous studies investigating heterochromatin reassembly after replication, how heterochromatin proteins affect centromere replication and the temporal relationship of replication and transcription at the centromere are still unclear. In this study, we examine the dynamics of the two chromodomain proteins, Swi6 and Chp1, in association with the centromere during mitosis. We further examine the effect of heterochromatin-associated mutants on replication timing.

Consistent with the early replication observed in *swi6*Δ *clr4*Δ double mutants [4], we find that *clr4*Δ as well as *chp1*Δ single mutants also replicate early, indicating that the pericentromeric domains are intrinsically early-replicating in the absence of the RITS complex or histone methylation. We also observe that mutations affecting the RDRC (*rdp1*Δ) or the Dicer protein *dcr1*Δ modestly delay replication timing, while *hrr1*Δ, which lacks a helicase that links RDRC and RITS, shows a Swi6-like phenotype. This suggests that a replication-refractory structure is still assembled in the RNAi mutant strains, despite the presence of Swi6. Finally, we show that delayed replication and delayed recruitment of DNA polymerase alpha in *swi6*Δ does not affect timing of RNA polymerase II recruitment. Together, these observations suggest several aspects of heterochromatin assembly may affect replication but the replisome is not required for recruiting RNA polymerase II of the pericentromeric repeats.

## Results

### Chp1 and Swi6 relocalize to the centromere at different times

Swi6 and Chp1 both bind to methylated histone H3K9 via their chromodomain [24]. Previous studies using chromatin immunoprecipitation analysis showed Swi6 is largely removed from the centromere in mitosis and into S phase, and returns in late S phase [8], [25]. We investigated whether this was also observed cytologically, using live cell analysis of asynchronously growing cells and GFP- tagged Swi6 or Chp1. Cells also expressed Sad1-DsRed, a spindle pole body marker (SPB) that is adjacent to the centromeres [26]. This allows us to monitor the timing of mitosis (based on the timing of SPB duplication and separation), and infer which Swi6 or Chp1 signal corresponds to the centromere.

**Figure 1:**
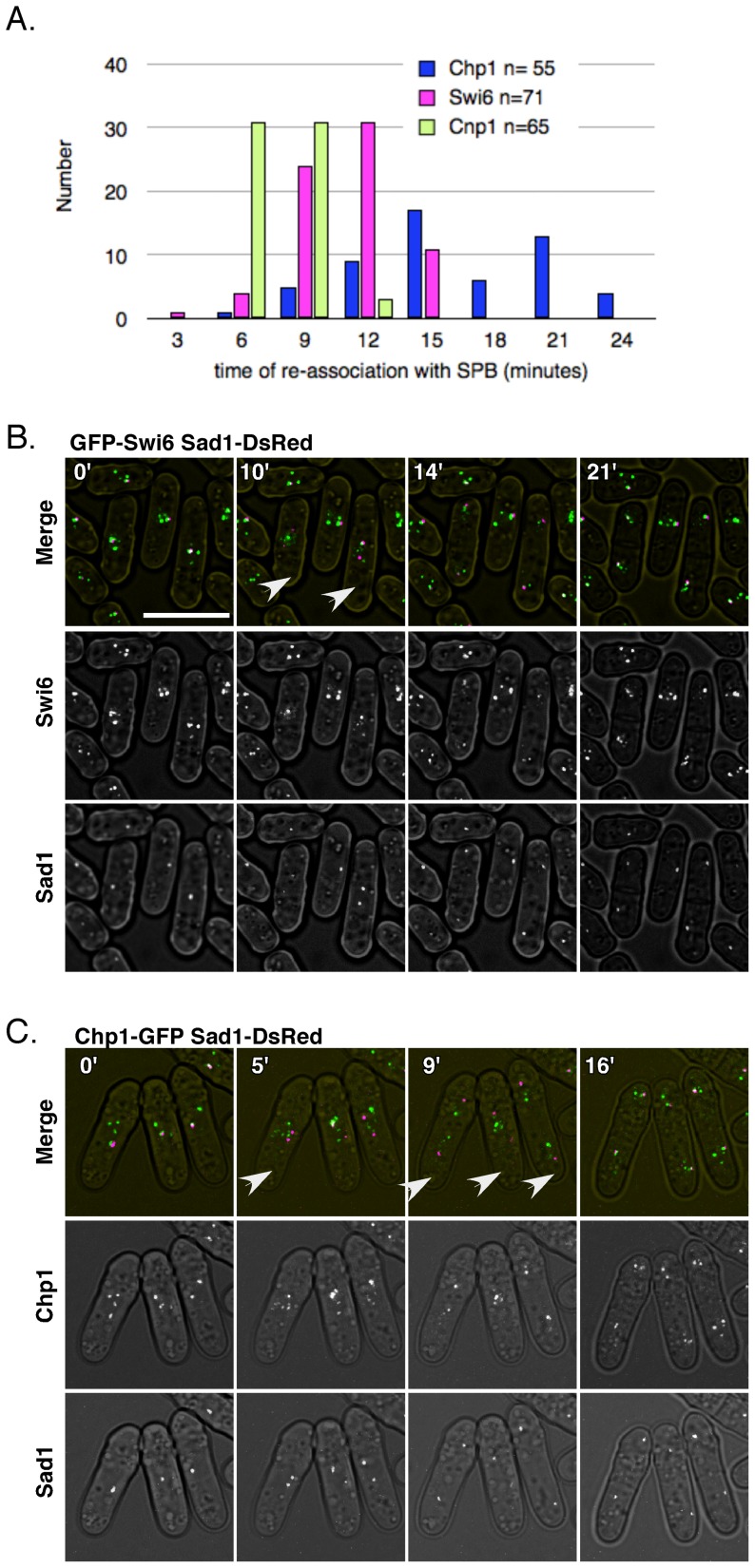
Swi6 and Chp1 each delocalize from the centromere between M and S phase. Asynchronously growing cells containing CFP-Cnp1 Sad1-DsRed (FY4229), GFP-Swi6 Sad1-DsRed (FY3665), or Chp1-GFP Sad1-DsRed (FY5911) were imaged every three minutes and the images were projected as described in materials and methods. A, quantitation of the data. The first frame in which spindle pole body duplication was observed was assigned as “0”. The first frame in which re-association of the GFP marked protein with the SPB occurred, and was maintained for at least three frames, was recorded. The re-association timing of CFP-Cnp1 (green), GFP-Swi6 (purple), or Chp1-GFP (black) with Sad1-DsRed is summarized in this distribution. Y axis, number of cells; X axis, time (minutes) after duplication of Sad1-DsRed, which is used to mark the beginning of M phase. Representative frames of GFP-Swi6, or Chp1-GFP are provided here B-C, respectively; selected original movies are provided in Movies S1, S2, S3. Arrows show cells with delocalization of the GFP signal from the SPB.

First, we established the timing of centromere association with the SPB during mitosis, by examining the centromere-specific histone CFP-Cnp1. As expected, CFP-Cnp1 briefly delocalizes from Sad1-DsRed at the time of SPB duplication, consistent with formation of the metaphase plate, but re-associates quickly as the centromeres move to the poles and the spindle elongates in early anaphase (Figure 1A, Movie S1) [27].

Next, we examined GFP-Swi6, a homologue of HP1 which binds histone H3K9me. Consistent with previous studies [28], we see 3–6 GFP foci, corresponding to the clustered centromeres, the mating type locus, and the telomeres. We observed that the GFP-Swi6 focus adjacent to the centromere delocalized at the time the Sad1-DsRed focus duplicated at the beginning of mitosis, consistent with entry into metaphase. However, in contrast to observations with Cnp1, GFP-Swi6 did not re-associate with the SPB until well after anaphase, (Figure 1A–B Movie S2). This is consistent with the timing of S phase, which occurs prior to septation, and agrees with molecular studies using ChIP [8], [25].

Finally, we examined the behavior of Chp1, another chromo-domain protein which is required to establish H3K9 methylation [29], [30]. Like Swi6, there are multiple Chp1 foci observed in the cell [31], [32] (Figure 1C). We observed that, similar to Swi6, the Chp1 foci adjacent to the Sad1-DsRed focus disappeared as the SPB duplicated, and returned just prior to septation (Figure 1A, C, Movie S3). We also observed that remaining scattered Swi6 and Chp1 foci remained intact, indicating that whatever mechanism disrupts Chp1 during mitosis does not affect these other regions of localization [29], [30].

Interestingly, the time of relocalization differs for Swi6 and Chp1. The mean time of re-association for Cnp1 was 7.71±1.75 min, compared to 10.98±2.5 for Swi6; in contrast, the association with Chp1 was 16.2±4.5 min; as suggested by the large standard deviation, there is a substantial variance in the distribution of Chp1 reassociation; but the distribution suggests its recruitment is delayed relative to Swi6 (Figure 1A). This is consistent with data suggesting that Swi6 interacts with core replication proteins to promote early replication in the centromere [15], [33], while Chp1 is recruited for heterochromatin establishment after replication [34].

### Centromere heterochromatin replicates early in *chp1*Δ and *clr4*Δ

**Figure 2:**
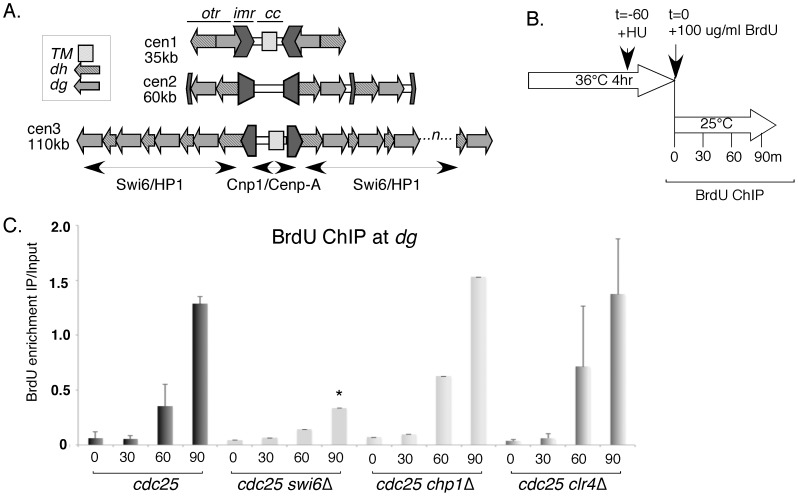
The centromere replicates early in *clr4*Δ or *chp1*Δ mutants. A, the structure of the three *S. pombe* centromeres. Repetitive sequences *dg* and *dh* in the outer repeats (*otr*) are present in slightly different organization in each centromere [66]. The length of *dg* or *dh* is around 4-6 kb. B, scheme of the experimental protocol. *cdc25-22* mutants were shifted to 36°C for 4 hours. One hour prior to release to 25°C, 10 mM of HU was added so that only early origins fire. Upon release, 100 µg/ml BrdU was added to label new DNA synthesis. C, incorporation of BrdU in the *dg* region was detected by BrdU enrichment, which was calculated by the ratio of IP versus Input by semi-quantitative PCR using primers #1536/1537 (dg). Three independent experiments were performed. Asterisks mark samples with BrdU signal significantly higher than the WT at 90 min with p<0.05 (Student's T test). The quality of synchronization is determined by flow cytomertry. There is no significant different among WT, *chp1*Δ and *clr4*Δ (Figure S1A).

Pericentromeric heterochromatin domains in *S.pombe* consist of a series of repeated motifs called *dg* and *dh;* these elements are interspersed with origins of DNA replication (Figure 2A). Previous work demonstrated that Swi6 is required for early replication timing in this region [4], [15]. Normal timing is restored in *swi6*Δ *clr4*Δ double mutants that lack the H3K9 methyltransferase [33]. We examined replication timing in single mutants *clr4*Δ and *chp1*Δ. Chp1 is part of the RITS complex required to recruit Clr4 and establish histone methylation. In *chp1*Δ or *clr4*Δ, Swi6 localization at the centromere is reduced. H3K9me is reduced in *chp1*Δ but absent in *clr4*Δ [34]–[36].

We examined replication timing by labeling cells with a deoxynucleotide analogue bromodeoxyuridine (BrdU) and examining its incorporation into the DNA by chromatin immunoprecipitation [15]. After extracting the genomic DNA, newly synthesized DNA was precipitated by an antibody specific to BrdU (BrdU ChIP) and the amount of BrdU enriched-DNA determined by semi-quantitative PCR. We synchronized the cells in G2 using a temperature sensitive allele *cdc25-22*. After shifting to restrictive temperature for 3 hours, we added 10 mM of Hydroxyurea (HU), which depletes the pool of deoxynucleotides so cells arrest in early S phase. After another hour at restrictive temperature, the cells were released into the cell cycle by shifting down to 25°C, at which time BrdU was added in the culture to label early replicating regions, still in the presence of HU (Figure 2B). This synchronization strategy prevents late origins from firing in wild type cells. Although high temperature attenuates heterochromatin silencing, a comparison of the behavior of Swi6 and recruitment of heterochromatin machinery during S phase using *cdc25* block and release or HU block-and-release shows no substantive differences [8], [25].

We observed that cell cycle progression from G2 to early S phase was similar in *swi6*Δ, *chp1*Δ, *clr4*Δ, and wild type and the present of HU was successfully blocked all the cells in S phase, as measured by flow cytometry (Figure S1A). Similar to previous observations [4], [15], the centromere replicated late in *swi6*Δ. In contrast, both *clr4*Δ and *chp1*Δ replicated with early timing, similar to wild type (Figure 2C). This suggests that the pericentromeric domains are intrinsically early-replicating, independent of Swi6, if H3K9me is absent or reduced.

### Centromere replication timing changes in *hrr1*Δ, *rdp1*Δ, and *dcr1*Δ

**Figure 3:**
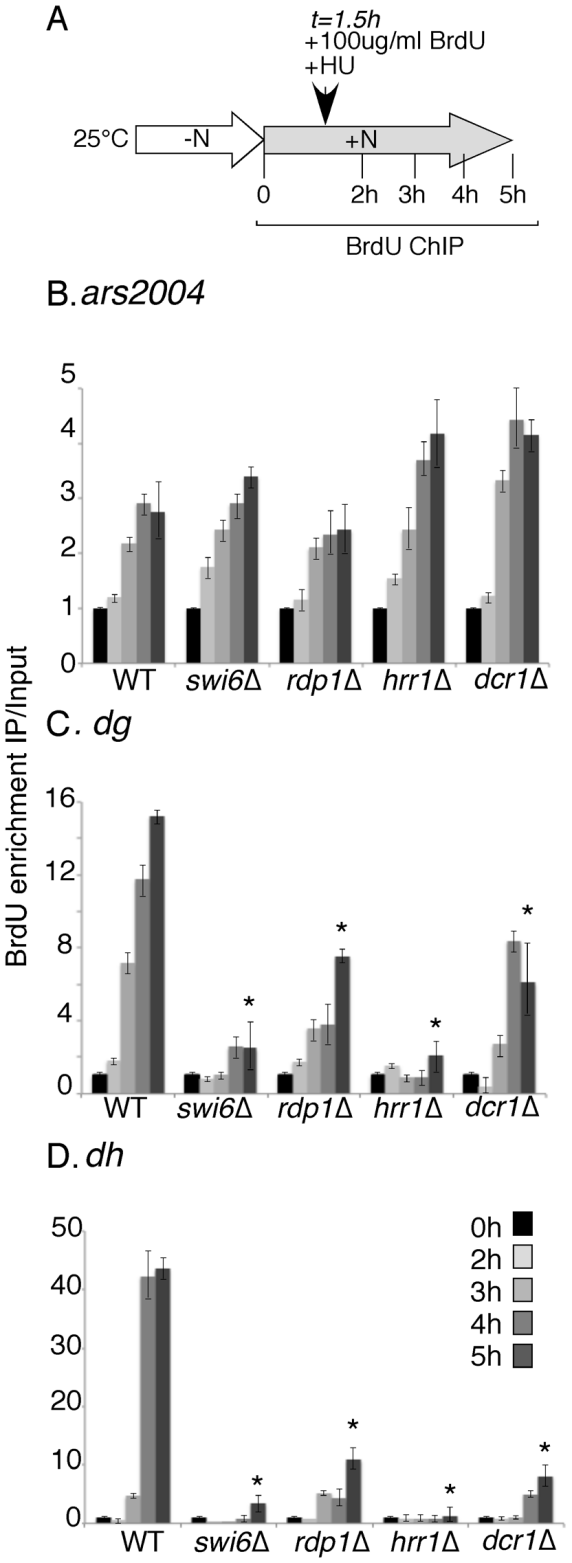
Centromere replication is delayed in RNAi mutants. A, schematic showing the experimental procedure. Cells were arrested in G1 by nitrogen depletion and released into the cell cycle by refeeding. BrdU and HU were added at 1.5 hours after release. B-D, BrdU ChIP in different mutants at the early origin *ars2004* (B), and the centromere repeats *dg* (C), and *dh* (D). BrdU enrichment was calculated by the ratio of IP versus Input by semi-quantitative PCR and at least two independent experiments were performed. Each mutant compared to its 0 timepoint. Asterisks mark samples with BrdU signal significantly higher than the WT at 6 h timepoint with p<0.05 (Student's T test). Primers: #1041/1042(dg), #1033/1034 (dh), and #1257/1258 (ars2004).

We next examined upstream components of the RNAi pathway, which is required to re-establish histone methylation following DNA replication [25], [35]. The double mutant of *dcr1*Δ *cdc25-22* grew very slowly with a very elongated cell morphology (data not shown). This synthetic interaction implies that Dcr1 affects cell cycle progression independent of centromere assembly, and is consistent with previous observations linking Dcr1 and Ago1 to cell cycle control [36]. This phenotype precluded the use of *cdc25* block and release to synchronize the cells. Therefore, we used nitrogen starvation to block the cells in G1, and released into early S phase in the presence of 10 mM HU. Cells were harvested at indicated time-points (Figure 3A), and we examined the incorporation of BrdU at an early origin, and the *dg* and *dh* repeats. Importantly, despite the growth defects, these mutants showed no change in euchromatic replication, indicating no changes in overall replication dynamics.

Dicer (*dcr1^+^)* is the ribonuclease that generates siRNAs for RITS-mediated heterochromatin assembly [37]. RNA-directed RNA polymerase (*rdp1* ^+^) generates double stranded RNAs for siRNA generation [37]. Hrr1 is an RNA helicase which links the RDRC and RITS complexes [38]. We compared *dcr1*Δ, *rdp1*Δ, and *hrr1*Δ to wild type and *swi6*Δ cells using the same protocol (Figure 3B–D). All three RNAi proteins are important for histone methylation and silencing at the centromere, and *a priori* would be expected to affect replication in the same way as *chp1*Δ or *clr4*Δ.

Unexpectedly, we found that replication timing in all three mutants was delayed. While *rdp1*Δ and *dcr1*Δ were not as severely delayed as *swi6*Δ, the replication delay in the *hrr1*Δ mutant is similar to that in *swi6*Δ. Thus, eliminating the RNAi pathway leads to late replication, and is therefore distinct from mutations in *clr4*Δ and *chp1*Δ.

### RNA polymerase II loads onto centromere and is independent of DNA replication machinery

Finally, we addressed whether replication timing affects transcription by examining the recruitment of DNA and RNA polymerases. Collisions between replication and transcription are known to be deleterious, and can lead to formation of ssDNA, breaks, or aberrant structures [39]. Recent work suggests that collisions between the replication and transcription machinery in the centromere contribute to genome instability [20]; this could influence origin usage or timing.

We synchronized cells in G2 using the temperature sensitive allele *cdc25-22* and released to S phase with BrdU at 21°C. The lower temperature was used to slow down the cell cycle for better resolution. At each time point, we monitored newly synthesized DNA by BrdU ChIP, and DNA polymerase alpha and RNA polymerase II loading by ChIP, and quantified using real-time PCR. For ChIP analysis, each time point was compared to its corresponding “mock” control. Cell cycle progression was monitored by septation index, which corresponds roughly with S phase and can be used as a metric for efficiency of synchronization. In this experiment, septation peaked with approximately 65% of cells at 130 min (Figure S1B).

**Figure 4:**
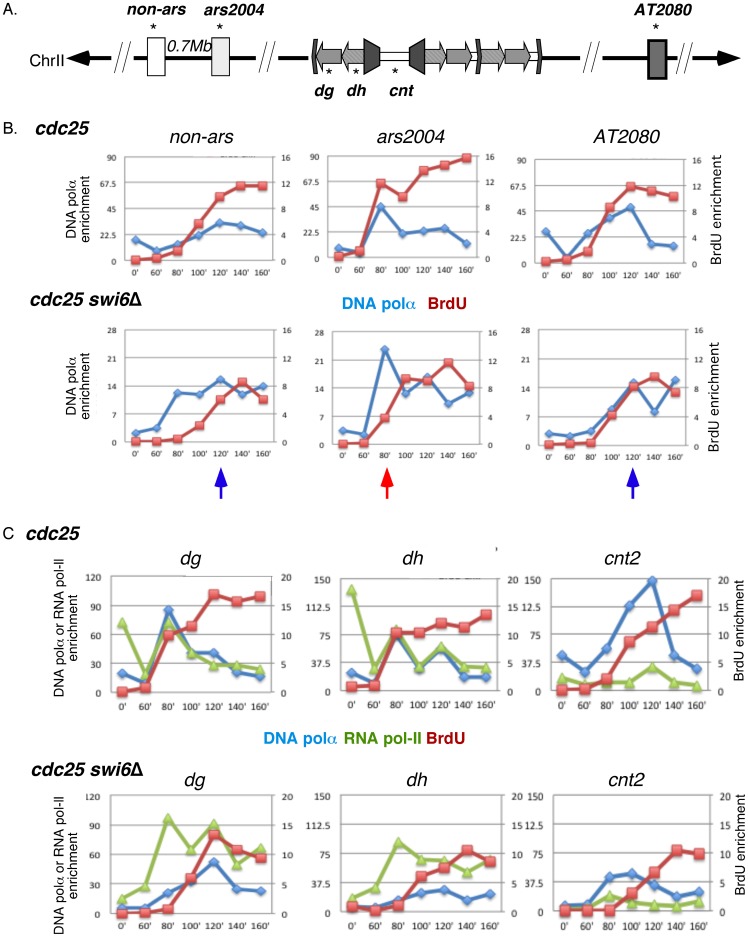
Replication timing and polymerases recruitment in wild type and *swi6*Δ. Cells were synchronized by *cdc25-22* block and release. Two independent experiments were performed and the representative result is presented. The signals were determined by quantitative real-time PCR. A, position of PCR probes used in this experiment. B–C, recruitment of DNA polymerase alpha (blue line), RNA polymerase II (green line) and incorporation of BrdU (red line) in euchromatin and the centromere, respectively. Left Y axis, DNA polymerase alpha or RNA polymerase II enrichment; right Y axis, BrdU enrichment. Red arrow, early replication time-point; blue arrow, late replication time-point. Primers: #1257/1258 (ars2004), #1265/1266 (non-ars), #875/876 (AT2080), #1628/1629 (dg), #1183/1184 (dh), and #879/880 (cnt2).

At the early euchromatic origin *ars2004*, we saw a peak of DNA polymerase alpha loading at 80 min, corresponding with the plateau of BrdU signal. The late origin *AT2080* and the *non-ars* control region showed a peak of DNA polymerase alpha loading and BrdU signal at 120 min, consistent with the end of S phase (Figure 4B). Thus, BrdU corresponds to DNA polymerase alpha recruitment.

Interestingly, in *swi6*Δ, DNA polymerase alpha enrichment was reduced compared to wild type, implying either less DNA polymerase alpha loading or less efficient ChIP in *swi6*Δ compared to wild type, even in this euchromatic region. At *ars2004*, although the peak of DNA polymerase association was at the same time as wild type, the accumulation of BrdU is slightly delayed (from 80 min to 100 min). At the *non-ars* region, DNA polymerase alpha peaked at 80 min, but the peak of BrdU enrichment was not until 140 min (Figure 4B). This suggests that DNA polymerase alpha recruitment precedes DNA synthesis in the *swi6*Δ mutants, and may reflect delay in initiation or fork progression in this euchromatin domain under these conditions. No delay in BrdU incorporation in the euchromatic region is observed using a nitrogen starvation/release protocol [15], so this may be an effect of the lower temperature used here.

We examined recruitment of RNA polymerase II compared to BrdU incorporation and DNA polymerase alpha binding in the centromere (Fig. 4C). RNA polymerase II enrichment at the centromere is very high at the initial time-point when cells are arrested 36°C. This has been observed previously; the pericentromeric heterochromatin silencing is temperature sensitive with the repeat transcripts accumulating even in wild type at 36°C [8], [40]. However, similar results for heterochromatin behavior during S phase are observed using *cdc25* block-and-release as in other forms of synchrony [8], [25]. In the absence of Swi6, there was no enrichment of RNA polymerase II at time 0, when the cells are arrested in G2 at the restrictive temperature.

After release into the cell cycle by reducing temperature, RNA polymerase II enrichment in wild type cells immediately decreased as expected [8], [25]. It returned to a peak at 80 min, corresponding exactly to the peak of DNA polymerase alpha and BrdU enrichment at *dg* and *dh* regions, and suggesting that transcription is coincident with early DNA synthesis.

It has been shown that no DNA synthesis occurs at the central core *cnt* domain in the present of HU [41] suggesting that *cnt* is a late replicating region. Consistent with this, we observed a peak of DNA polymerase alpha and BrdU at 120 min, when late euchromatin origins fire. Interestingly, there was also small peak of RNA polymerase II observed at 120 min in this domain, consistent with evidence that it is expressed at a low level [40].

In *swi6*Δ cells, DNA synthesis and polymerase alpha loading were both delayed relative to wild type, until 100 –120 min (Figure 4), consistent with previous observations ([4], [15], Fig. 2). However, RNA polymerase II still loaded onto *dg* and *dh* regions at 80 min, an early time point. Thus, the RNA polymerase II recruitment is independent of the replisome in a *swi6*Δ mutant.

## Discussion

The timing of replication origin firing is influenced by chromatin structure and histone modifications. In most organisms, heterochromatin is replicated late in S phase [42], [43]. Heterochromatin in fission yeast is typically defined by chromodomain-mediated association of the Swi6/HP1 protein with methylated histone H3K9 [44]. In contrast to other heterochromatin domains, the *S. pombe* pericentromere undergoes Swi6-dependent early replication [5], [6], [41]. Swi6/HP1 is known to interact with replication proteins including the DDK kinase [4], [16], Cdc18^Cdc6^ [15], [45], ORC [46], [47] (PCL and SLF, unpublished results) and PCNA [23], [48]–[50]. While centromeres in Drosophila are also late replicating, there are regions in the fly that undergo HP1-dependent early replication in flies, similar to observations in fission yeast [51]. These are often (but not always) associated with transcribed genes embedded in repetitive sequences and bound by HP1 [52].

Previous studies have shown that the requirement for Swi6 for early replication can be bypassed by tethering the DDK regulatory subunit Dfp1 to a chromodomain, to recruit it to H3K9 methylated heterochromatin [4]. This is consistent with data suggesting that DDK association affects replication origin efficiency [17]. Together, these observations suggest that the impact of Swi6 on replication timing is mediated by its ability to recruit otherwise limiting replication factors to this region.

Most Swi6 is dissociated from the pericentromere during mitosis [8], [25]. This transient delocalization allows a brief window of transcription that generates siRNAs; these program an RNAi mechanism that targets the methylation machinery back to the centromere to re-establish histone methylation and silencing [53].

Using microscopy in live cells, we observed that the bulk of Swi6 delocalizes during mitosis from the pericentromeric regions and reassembles shortly after anaphase. This is consistent with delocalization being driven by H3S10 phosphorylation during mitosis, which occurs genome-wide [8], [25]. We observed similar delocalization of Chp1, a chromodomain protein that is part of the RITS complex required for methylation of the pericentromeric heterochromatin. As seen previously [31], [38], [54], we observe multiple foci associated with Chp1-GFP in fission yeast cells. However, only the focus at the centromere, associated with spindle pole component Sad1, disperses during mitosis. While molecular assays show Chp1 associates with telomeres and mating type loci [31], cytological experiments have not confirmed that these regions are associated with the additional Chp1 puncta we observe, although this seems likely. Transcription-coupled acetylation of histones during S phase is proposed to dislodge the high-affinity binding of Chp1 to H3K9me at the centromere, allowing Swi6 to bind in its place [34], [55]. Because transcription does not occur at the telomeres and silent mating type loci, we suggest that Chp1 is likely to remain associated with H3K9me in those domains during S phase.

Importantly, our video microscopy data suggest that some Swi6 is recruited back to the centromere prior to Chp1. This may reflect the recruitment of Swi6 by replication proteins including ORC, DDK (Hsk1) kinase, and Cdc18^Cdc6^, in addition to binding to H3K9me ([4], [15] unpublished observations). Close inspection of molecular data in [8], [22] suggest that there may be two stages of Swi6 recruitment; a small association early in S phase, and a substantial association later in S phase as the H3K9me mark is fully re-established. This would be consistent with recent data suggesting that Swi6 also functions upstream of RNAi [14]. The late S phase association of Swi6 may be the stage at which there is a transition from Chp1 to Swi6 as suggested in [34], [55].

The late replication associated with *swi6*Δ can be suppressed by loss of the methyltransferase Clr4 [4]. We showed that *clr4*Δ single mutants, which disrupt methylation and therefore Swi6 binding, also replicate early. This indicates that origins in the pericentromere are intrinsically early-replicating, and shows that Swi6 is not required for early replication if H3K9 methylation is absent.

One model suggests that if RNAi is required for histone methylation, mutants lacking in RDRC or Dicer should also be early replicating. Instead, we find that *rdp1*Δ and *dcr1*Δ mutants have delayed replication, though not as severe as *swi6*Δ, while replication in *hrr1*Δ mutants is just as late as *swi6.* Hrr1 is an RNA helicase that couples RITS and RDRC; it is required for silencing and for methylation [38]. Recent studies suggest that Hrr1 and the RDRC bind directly to Swi6 via a mediator protein, Ers1 [12], [14]. It is possible that the severe replication delayed in *hrr1*Δ is due to the missing of Swi6, or the present of Chp1, or the combination of both.

A recent study in fission yeast suggests that in the absence of Dcr1, there is a failure of transcription termination in the centromere, leading to collisions between RNA polymerase and the DNA replication machinery and resulting in DNA damage [20]. Collision between replication and transcription machineries is known to contribute to disruptions in genome stability (rev. in [56]). Such collisions are reduced in *swi6*Δ cells [20], which would be consistent with their delayed initiation of replication ([4], [15], this work). This effect is likely to be minimal, however, because *swi6*Δ mutants have only a modest silencing defect, and do not accumulate substantial transcripts [57], [58]. We observe RNA polymerase II is recruited with wild type timing in *swi6*Δ mutant. Other studies have suggested that RNA polymerase II recruitment is linked to leading strand DNA synthesis in *swi6^+^* cells [19], but that may be a redundant mechanism since we see recruitment of RNA polymerase II prior to DNA polymerase in *swi6*Δ.

In *clr4*Δ mutants, there is also evidence that collisions between transcription and replication machinery are reduced [20]. However, this isn't due to delayed replication, because we show that *clr4*Δ mutants replicate early. Importantly, these data show us that early replication and increased transcription are compatible, and we see no obvious correlation between levels of transcripts and replication timing. Thus, it cannot be increased transcription *per se* that leads to the delayed replication in the RNAi mutants.

Recently it was shown that Ers1 and Hrr1 proteins are recruited to the chromatin in *chp1*Δ but not in *swi6*Δ or *clr4*Δ cells [14]. Since *swi6*Δ and *clr4*Δ have different effects on replication timing, but both prevent RDRC recruitment, we conclude that RDRC recruitment is not related to replication timing. Likewise, although centromere silencing is impaired in *rdp1*Δ, *hrr1*Δ, or *dcr1*Δ cells, there is still residual H3K9me and Chp1 at this region in RNAi mutants [38], [59], consistent with evidence that there is RNAi-independent maintenance of heterochromatin [60]–[62]. And, there is residual H3K9me present in *chp1*Δ mutants [14], [54], [63]. The presence of residual H3K9 methylation in both early (*chp1*Δ*)* and late (*hrr1*Δ*)* replicating mutants suggests that it is not H3K9 methylation that affects replication timing.

Chp1 binding to the centromere is seen in the absence of Swi6 and RNAi proteins but requires Ers1 and Clr4 [14], [29], [60]–[61]. This is consistent with our observation that *swi6*Δ, *hrr1*Δ, *rdp1*Δ, and *dcr1*Δ are late replicating (some Chp1 is present), but *clr4*Δ is early-replicating (Chp1 is absent). Thus, we propose that early Swi6 assembly in the centromere, perhaps assisted by its association with replication factors, counteracts negative effects on replication associated with Chp1 binding (which has higher binding affinity to H3K9me than Swi6; [55]). Swi6 association with DDK, the replication initiation kinase, is required for early replication unless clr4Δ is also missing (Hayashi 2009), which suggests that DDK is required specifically to counter a Clr4-dependent event. Since artificially tethering DDK to the chromatin overcomes this effect [4], there may be a direct role for the kinase in countering the inhibitory effect of Chp1. Interestingly, we do not observe delocalization of Chp1 from other heterochromatin domains that are known to replicate late. Future work will be required to determine whether Chp1 is recruited to the centromere earlier in *swi6*Δ mutants or whether its inhibition of early replication timing in the centromere is due to its unopposed binding in this domain.

## Materials and Methods

### Yeast Growth and Strains

Yeast strains were constructed using standard protocols [64] and are listed in Table S1. For BrdU labeling, cells expressed Herpes Simplex virus thymidine kinase *(hsv-tk^+^)* and the hENT nucleoside transporter [65]. For temperature shift experiments, cells were grown in EMM medium with necessary amino acid supplements at 25°C until OD 0.3 (3.75×10^6^ cells/ml). Cells were then shifted to 36°C for 4 hours and cooled down in an ice bath to 21°C. Time points were collected as indicated and the quality of synchrony was monitored by counting the appearance of septation in 200 cells by hemocytometer using phase microscopy. For synchrony by nitrogen starvation, strains competent to take up exogenous thymidine were used. Cells were grown to OD_600_ 0.3–0.5 and nitrogen starved for 16 hours at 25°C. Cells were released by re-feeding with nitrogen-containing medium at 25°C.

### ChIP Analysis

ChIP analysis was carried out as described [12] with minor modifications. When ChIPing DNA polymerase alpha-Flag (M2, Sigma) and RNA polymerase II (8WEG, Covance), cells were crosslinked with 1% formaldehyde for 30 minutes at 21°C.

### BrdU ChIP

Cells were synchronized by 4 hours incubation at 36°C and released at 21°C, at which time 100 ug/ml bromodeoxyuridine (Sigma) was added into the cell culture. BrdU ChIP analysis was performed as previously described [15]. All the primers used in this study are listed in Table S2.

### Live Cell Analysis

Cells were grown in supplemented EMM medium and plated on agarose pads [66]. Images were acquired with a DeltaVision Core (Applied Precision, Issaquah, WA) microscope using a 60x N.A. 1.4 PlanApo objective lens and a 12-bit Photometrics CoolSnap HQII CCD. The system x-y pixel size is 0.109 µm. softWoRx v4.1 or v5.0 (Applied Precision, Issaquah, WA) software was used at acquisition. Excitation illumination was from a Solid-state illuminator, GFP-Swi6 was excited and detected with a 475/28, 525/50 filter set (excitation intensity attenuated to 5%) and a 80 ms exposure (Chp1-GFP used similar filters and a 250 ms exposure); DsRed was excited and detected with a 575/25, 632/60 (excitation intensity attenuated to 10%) filter set and a 200 ms exposure. A suitable GFP/RFP polychroic mirror was used. Fourteen 0.4 µm serial z-sections were captured for Swi6 and Cnp1 (ten for Chp1). CFP-Cnp1 used a 438/24, 470/24 filter set, intensity 5% and 150 ms exposure (CFP was multiplexed with the red channel with a RFP/CFP mirror). Image stacks were acquired at 180 s intervals. 3-D stacks were deconvolved with softWoRx, and images were maximum intensity projected for presentation. Images were contrast adjusted using a histogram stretch on a similar scale and gamma for comparability. Brightfield reference images were also acquired. Temperature was maintained at 30°C using a WeatherStation (Applied Precision).

To determine the timing of reassociation of the GFP/CFP-marked proteins with the spindle pole bodies, we examined the deconvolved projections. We marked the first frame in which SPB duplication was observed (in all cases, this was accompanied by dissociation of the GFP/CFP protein). We then determined the first time-point following in which (a) at least one SPB showed association with the GFP/CFP protein and (b) this association lasted for at least three ensuing frames.

### Real-time PCR

384-well quantitative PCR were performed on ABI7900 machine by SDS 2.3 software. Fold enrichment of each primer set was calculated by Pfaffl method [67]. The fold enrichment of ChIP samples was relative to the mock immunoprecipitation. For each primer set, ΔCt was obtained by Ct^immunoprecipitation^ minus Ct^Input^. The fold enrichment of BrdU ChIP was relative to the input signal. Primer sequences are provided in Table S2.

## Supporting Information

Figure S1 **Quality of cell cycle synchronization is examined by either flow cytometry (A,** **Figure 2** **), or septation index (B,** **Figure 4** **).**
(TIF)

Movie S1 **Time-lapse movie of cells with CFP-Cnp1 and Sad1-DsRed.** The imaging procedure is described in materials and methods.
(MOV)

Movie S2 **Time-lapse movie of cells with GFP-Swi6 and Sad1-DsRed.** The imaging procedure is described in materials and methods.
(MOV)

Movie S3 **Time-lapse movie of cells with Chp1-GFP and Sad1-DsRed.** The imaging procedure is described in materials and methods.
(MOV)

Table S1 **Strains list.** Strains used in this study.
(PDF)

Table S2 **Primers list.** Primers used in this study.
(PDF)
