# Correction: Mutations Disrupting Histone Methylation Have Different Effects on Replication Timing in *S. pombe* Centromere

**DOI:** 10.1371/annotation/5d4b57ee-38c3-4979-b1bd-b257302550bc

**Published:** 2013-11-12

**Authors:** Pao-Chen Li, Marc D. Green, Susan L. Forsburg

The number of the National Science Foundation grant is incorrect. The correct grant number is: NSF MCB 0743448. 

