# Supplementary figures and images for: Mutations Disrupting Histone Methylation Have Different Effects on Replication Timing in S. pombe Centromere
Source: PLoS One. 2013 May 1;8(5):e61464. doi: 10.1371/journal.pone.0061464 (PMC3641051; doi:10.1371/journal.pone.0061464)

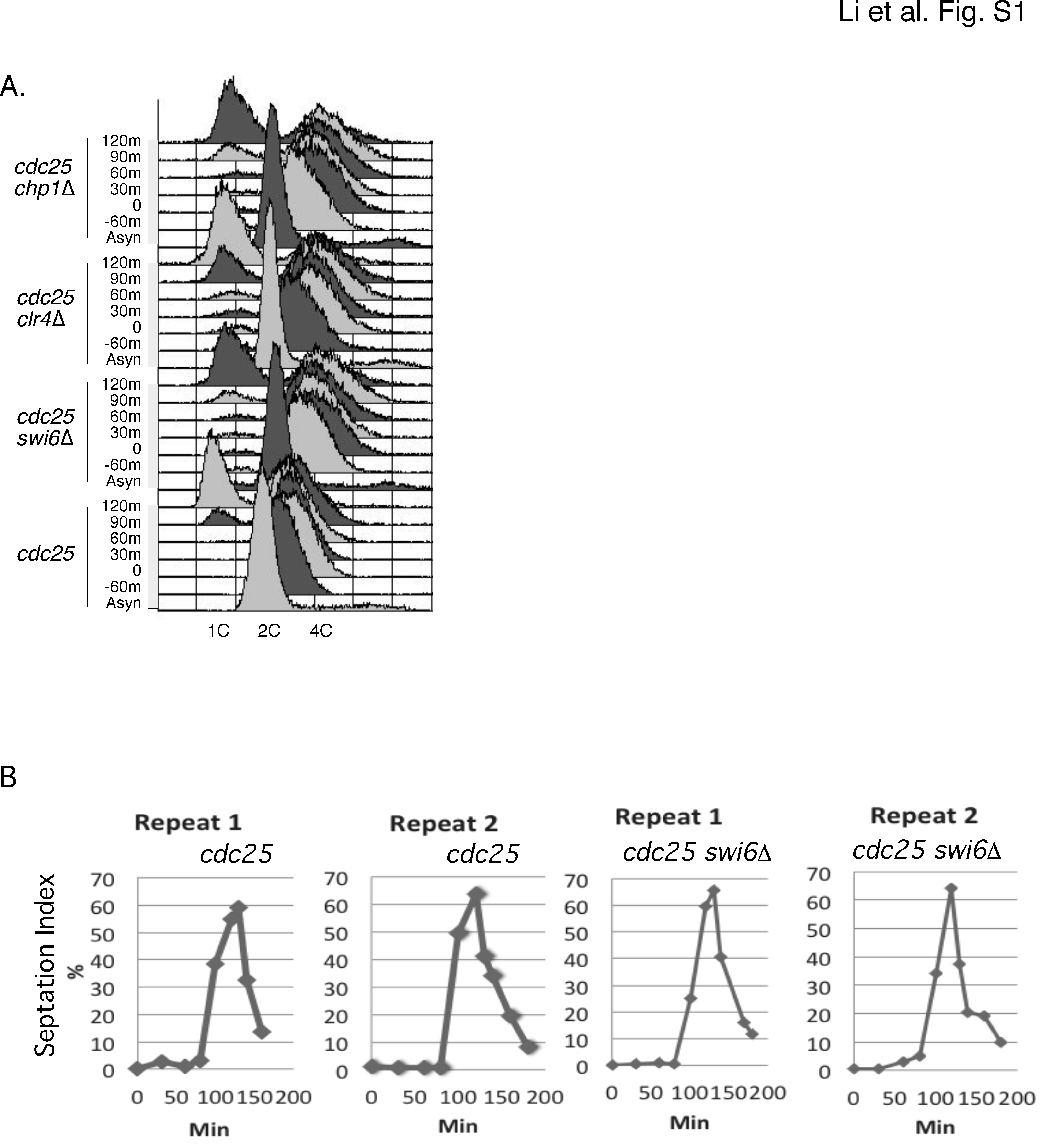

Supplement: Figure S1 — Quality of cell cycle synchronization is examined by either flow cytometry (A, Figure 2 ), or septation index (B, Figure 4 ). (TIF) [file pone.0061464.s001.tif]
