# Supplementary material for: Mutations Disrupting Histone Methylation Have Different Effects on Replication Timing in S. pombe Centromere
Source: PLoS One. 2013 May 1;8(5):e61464. doi: 10.1371/journal.pone.0061464 (PMC3641051; doi:10.1371/journal.pone.0061464)
Supplement: Table S1 — Strains list. Strains used in this study. (PDF) [file pone.0061464.s005.pdf]

Table S1. Strains list.

Strain Number	Genotype	Source
FY2318	*h+ cdc10-V50 leu1-32::hENT1-leu1+(pJAH29) his7-366::hsv-tk-his7+(pJAH31) ade6-M216 ura4-D18*	Li et al., 2011
FY3665	h90 swi6Δ::his1+ sad1+::DsRed-LEU2+ leu1::[Leu+-nmt-GFP-swi6] lys1	This study
FY3812	*h+ cdc10-V50 swi6Δ::ura4+ leu1-32::hENT1-leu1+(pJAH29) his7-366::hsv-tk-his7+(pJAH31) ade6-M216 ura4-D18*	Li et al., 2011
FY4223	h- rdp1Δ::natR leu1::hENT-Leu1+ his7-366::hsv-tk-his7+ uraD18 i mr1R::ura4+ oriI	This study
FY4224	h- hrr1Δ::natR leu1::hENT-Leu1+ his7-366::hsv-tk-his7+ ade6-M216 ura4D18	This study
FY4228	h- dcr1Δ::natR leu1::hENT-Leu1+ his7-366::hsv-tk-his7+ ade6-M216 ura4D18	This study
FY 4229	h? ura4-D18 CFP-cnp1::kanMX rad22:YFP-kanMX sad1::DsRed-Leu2+	This study
FY4255	h- cdc25-22 leu1::hENT-Leu1+ his7-366::hsv-tk-his7+ ura4? ade6-M210	This study
FY4487	h- cdc25-22 mrc1Δ::ura4+ leu1-32::hENT1-leu1+(pJAH29) his7-366::hsv-tk-his7+(pJAH31) ura4-D18 ade6-M210	This study
FY4488	h- cdc25-22 mrc1Δ::ura4+ leu1-32::hENT1-leu1+(pJAH29) his7-366::hsv-tk-his7+(pJAH31) rad11myc::KanMX ura4-D18 ade6-M210 (rad11=ssb1)	This study
FY4548	h+ cdc25-22 polα-FLAG2::ura4+ ade6-M216 ura4-D18 leu1-32::[hENT leu1+] his7-366::[hsv-tk his7+]	This study
FY4574	h+ cdc25-22 swi6Δ::kanMX leu1-32::hENT1-leu1+(pJAH29) his7-366::hsv-tk-his7+(pJAH31) ura4-D18 ade6-M210 his3-D1?	This study
FY4664	h? cdc25-22 clr4Δ::ura4+ leu1-32::hENT1-leu1+(pJAH29) his7-366::hsv-tk-his7+(pJAH31) (rad11myc::KanMX)? ura4-D18 ade6-M216/M210? (rad11=ssb1)	This study
FY4710	h+ cdc25-22 chp1Δ::kanMX6-Bioneer leu1::hENT-Leu1+ his7-366::hsv-tk-his7+ ura4? ade6-M210	This study
FY5321	h+ cdc25-22 swi6Δ::kanMX polα-FLAG2::ura4+ ade6-M210 leu1::[hENT leu1+] his7-366::[hsv-tk his7+] ura4-D18	This study
FY5911	h+ chp1::chp1-eGFP-KanMX sad1+::DsRed-LEU2 leu1-32 ura4-D18 ade6-M210/216 can1-1?	This study
