# Supplementary material for: Mutations Disrupting Histone Methylation Have Different Effects on Replication Timing in S. pombe Centromere
Source: PLoS One. 2013 May 1;8(5):e61464. doi: 10.1371/journal.pone.0061464 (PMC3641051; doi:10.1371/journal.pone.0061464)
Supplement: Table S2 — Primers list. Primers used in this study. (PDF) [file pone.0061464.s006.pdf]

Table S2. Primers list.

Primer number	Sequence	Source
875	CGAACAACAGGCTTGGTTAGAA	Hayashi et al., Gen Dev (2007) 26: 1327-39
876	GAAGTACGGACTTGTTTCGATTCC	Hayashi et al., Gen Dev (2007) 26: 1327-39
879	GGTTTTATGATTTTGGCGAAGTG	Hayashi et al., Gen Dev (2007) 26: 1327-39
880	CGACAAGCGTGGATTTATTTTATG	Hayashi et al., Gen Dev (2007) 26: 1327-39
1033	GTAAGTATGAGCAACTGGCG	Gomez et al., MCB (2005) 20: 8887-903
1034	GGAACAAATCAGGAAACCGAG	Gomez et al., MCB (2005) 20: 8887-903
1183	CCATATCAATTTCCCATGTTCC	Kloc et al., Curr Biol (2008) 28: 490-5
1184	CATCAAGCGAGTCGAGATGA	Kloc et al., Curr Biol (2008) 28: 490-5
1257	CGGATCCGTAATCCCAACAA	Nitani et al, PNAS (2008) 105: 12973-8
1258	TTTGCTTACATTTTCGGGAACTTA	Nitani et al, PNAS (2008) 105: 12973-8
1263	GTTACAATGGTTTCCAAACTTTTAG	Nitani et al, PNAS (2008) 105: 12973-8
1264	CATCGATTATTGTTTGCACGTT	Nitani et al, PNAS (2008) 105: 12973-8
1265	AAATATGGCGATCCAGGAGATG	Nitani et al, PNAS (2008) 105: 12973-8
1266	GCTTAACGTGCGCACAGACA	Nitani et al, PNAS (2008) 105: 12973-8
1536	TTTTCAGCGAGACATGTACC	Gomez et al., MCB (2005) 20: 8887-903
1537	TCATAAAGCAACACTGGGTG	Gomez et al., MCB (2005) 20: 8887-903
1624	AGAGCATGGTGGTGGTTATGG	PNAS (2009) 106: 1163-1168.
1625	TTTGGCGACTAAACCGAAAGC	PNAS (2009) 106: 1163-1168.
1628	CCGCAGTTGGGAGTACATCATTC	PNAS (2009) 106: 1163-1168.
1629	ACAGCACTCAACAACAGTCTTGG	PNAS (2009) 106: 1163-1168.
